# Epidemiological Characteristics, Indications, and Clinical Outcomes of Pediatric Tracheotomy in Serbia

**DOI:** 10.3390/children13060797

**Published:** 2026-06-09

**Authors:** Katarina Stanković, Vladan Šubarević, Mladen Novković, Sandra Šipetić-Grujičić, Ivana Fajertag, Slađana Vasiljević, Jadranka Maksimović, Isidora Vujčić

**Affiliations:** 1 Institute for Health Care of Mother and Child of Serbia “Dr Vukan Čupić”, 11070 Belgrade, Serbia; dr.kattja.stankovic@gmail.com (K.S.); vladan.subarevic@imd.org.rs (V.Š.); mladen.novkovic@imd.org.rs (M.N.); ivana.fajertag@imd.org.rs (I.F.); sladjana.vasiljevic@imd.org.rs (S.V.); 2 Institute of Epidemiology, Faculty of Medicine, University of Belgrade, Višegradska 26a, 11000 Belgrade, Serbia; sandra.grujicic@med.bg.ac.rs (S.Š.-G.); jadranka.maksimovic@med.bg.ac.rs (J.M.)

**Keywords:** pediatric tracheotomy, respiratory support, airway obstruction, decannulation, complications, outcomes

## Abstract

**Highlights:**

**What are the main findings?**

- Pediatric tracheotomy is increasingly performed for long-term respiratory support, with neurological disorders as the leading indication.
- Children with airway obstruction are younger, require more urgent procedures, and have significantly higher decannulation success rates than those needing respiratory support.

**What are the implications of the main findings?**

- Outcomes of pediatric tracheotomy are strongly influenced by underlying conditions, emphasizing the need for individualized, multidisciplinary care.
- Lower decannulation rates in patients requiring respiratory support highlight the need for improved long-term management strategies and follow-up protocols.

**Abstract:**

**Background/Objectives**: Pediatric tracheotomy has evolved from an emergency procedure for acute infections to a planned intervention for chronic conditions requiring prolonged airway support. This study aims to describe the clinical characteristics, indications, and outcomes of pediatric tracheotomy over a 21-year period at a tertiary care center. **Methods**: A retrospective observational case series was conducted, including 246 pediatric patients (0–18 years) who underwent tracheotomy between 2004 and 2024. Data were collected from medical records and included demographics, indications, procedural details, complications, decannulation, and mortality. Patients were categorized into airway obstruction (AO) and respiratory support (RS) groups. Statistical analyses were performed using the Mann–Whitney U test, Chi-square and Fisher’s exact test. **Results**: A significant increase in tracheotomy procedures was observed over time. Respiratory support was the predominant indication (75.2%), mainly due to neurological disorders, while airway obstruction accounted for 24.8%. Patients in the AO group were significantly younger and more likely to undergo urgent procedures (*p* < 0.001). Complication rates were comparable between groups (AO 16.4% vs. RS 21.1%; *p* = 0.295). Decannulation was significantly more successful in the AO group (16.4% vs. 5.4%; *p* = 0.012). Mortality did not differ significantly between groups and was associated with underlying comorbidities. **Conclusions**: Pediatric tracheotomy is increasingly performed for chronic respiratory support. While procedural safety is high, outcomes vary by indication, with better decannulation rates in airway obstruction cases. Multidisciplinary, individualized management is essential for optimizing patient outcomes.

## 1. Introduction

Tracheotomy remains an indispensable procedure in contemporary pediatric airway management, providing a definitive airway in children with upper airway obstruction (AO), chronic respiratory failure, and prolonged respiratory support (RS) [1,2]. Over recent decades, the epidemiology of pediatric tracheotomy has undergone a substantial transition, shifting from predominantly acute infectious indications toward chronic and complex conditions associated with advances in neonatal and pediatric intensive care, improved survival of premature infants, and increasing prevalence of children with medical complexity [2,3,4].

In current clinical practice, pediatric tracheotomy is most frequently performed in patients with severe neurological impairment, congenital or acquired airway abnormalities, chronic pulmonary disease, and long-term ventilator dependence [2,5,6,7]. As survival rates among critically ill children continue to improve, the population of tracheotomized children requiring prolonged multidisciplinary care and long-term support has expanded considerably, creating significant clinical, organizational, and socioeconomic challenges for healthcare systems [5,6].

Despite advances in surgical technique, perioperative management, intensive care, and home ventilation programs, pediatric tracheotomy remains associated with substantial morbidity and prolonged healthcare dependence [8,9]. Both early and late complications remain clinically relevant and include accidental decannulation, cannula obstruction, granuloma formation, local infection, tracheal stenosis, and persistent tracheocutaneous fistula [1,9,10]. Furthermore, although procedure-related mortality has declined substantially, overall mortality among tracheotomized children remains closely associated with the severity of underlying comorbid conditions and chronic respiratory failure [8,11].

Long-term outcomes following pediatric tracheotomy are strongly influenced by the primary indication for the procedure. Previous studies have demonstrated significantly higher decannulation rates and shorter cannulation duration among children undergoing tracheotomy for airway obstruction compared with patients requiring chronic respiratory support (RS) secondary to neurological or cardiopulmonary disease [3,7,12]. Nevertheless, considerable heterogeneity in reported outcomes persists across published cohorts, likely reflecting differences in institutional expertise, referral patterns, healthcare infrastructure, availability of pediatric intensive care resources, and decannulation strategies [7,13].

Current evidence regarding pediatric tracheotomy is derived predominantly from retrospective single-center series and multicenter studies conducted in high-income healthcare systems [4,6,13]. In contrast, long-term epidemiological data from Southeast European tertiary pediatric institutions remain limited. Consequently, regional variations in indications, clinical management, complication profiles, decannulation practices, and mortality outcomes remain insufficiently characterized within this healthcare context. In addition, relatively few studies have directly compared demographic and clinical outcomes between children undergoing tracheotomy for airway obstruction and those requiring respiratory support within the same institutional cohort [3,7,12].

Given these limitations, further investigation from underrepresented healthcare settings is warranted to improve understanding of the epidemiological and clinical characteristics of pediatric tracheotomy. The present study aimed to evaluate indications, epidemiological characteristics, complications, decannulation outcomes, and all-cause mortality associated with pediatric tracheotomy over a 21-year period at a tertiary pediatric referral center in Serbia. Particular emphasis was placed on comparative analysis between children undergoing tracheotomy for AO and those requiring RS in order to better define differences in patient characteristics, procedural outcomes, and long-term clinical course between these distinct patient populations.

## 2. Materials and Methods

### 2.1. Study Design and Population

This observational study presents a single-center case series of pediatric patients who underwent tracheotomy at the Mother and Child Health Care Institute of Serbia “Dr Vukan Čupić” between 1 January 2004, and 31 December 2024. Institutional review board approval was obtained prior to data collection. A total of 264 consecutive patients aged 0–18 years were identified through a comprehensive retrospective review of electronic medical records (Heliant system, Heliant d.o.o., Belgrade, Serbia) and physical archives. Eighteen patients were excluded due to insufficient documentation or loss to follow-up, resulting in a final study sample of 246 patients. Only the first tracheotomy performed in each patient during the study period was included in the analysis. Repeat tracheotomies or revision procedures in previously decannulated patients were not counted as separate cases in order to avoid duplication bias. Patients transferred from other institutions were included only if complete perioperative and follow-up data were available in our records. Conversely, patients transferred out of our institution shortly after tracheotomy were excluded from outcome analyses if reliable long-term follow-up information could not be obtained.

### 2.2. Data Collection

Data were extracted through a detailed review of operative reports, clinical progress notes, and discharge summaries. The following variables were collected: demographic characteristics (age at tracheotomy, sex); clinical parameters (primary indication, comorbidities); procedural details (urgency status, intraoperative ventilation status); and postoperative outcomes (complications, decannulation success, and mortality). All procedures were performed by attending pediatric otolaryngologists in accordance with a standardized institutional protocol. Insufficient documentation was defined as the absence of essential clinical data regarding indication, operative details, or postoperative outcomes. Loss to follow-up was defined as the absence of any subsequent clinical records after hospital discharge, which precluded reliable assessment of decannulation status or survival outcomes.

### 2.3. Surgical Technique

Patients were positioned with mild neck extension, and the surgical field was prepared using an antiseptic solution. Local anesthesia with 1% lidocaine and 1:100,000 epinephrine was infiltrated at the incision site. A vertical midline incision was made extending from the inferior border of the cricoid cartilage to the sternal notch. After midline dissection through the strap muscles, the anterior tracheal wall was exposed. The tracheotomy was typically created between the second and third or third and fourth tracheal rings. An age-appropriate Tracoe™ tracheostomy tube (Tracoe medical GmbH, Nieder-Olm, Germany) was inserted under direct visualization and secured with neck ties. Although the fundamental operative principles remained consistent throughout the study period, minor variations in surgical technique occurred over time according to surgeon preference, patient age, anatomical considerations, and evolving institutional practice. These included differences in tracheostomy tube selection, selective use of stay sutures, and methods of stomal maturation. No major institutional changes to the standard pediatric tracheotomy technique were introduced during the study period.

### 2.4. Classification of Tracheotomy Indications

Indications were categorized into two principal groups:

1. Airway obstruction, including laryngotracheal pathologies such as stenosis or malacia, and craniofacial anomalies.
2. Respiratory support, including chronic pulmonary failure, cardiopulmonary diseases, neurological disorders, genetic syndromes associated with respiratory compromise, and prolonged ventilator dependence following failed extubation.

### 2.5. Postoperative Complications and Decannulation

Postoperative complications were classified as early (<14 days postoperatively) or late (≥14 days postoperatively). Early complications included hemorrhage requiring intervention, surgical site infection, pneumomediastinum or pneumothorax, tube obstruction or displacement, and creation of a false passage. Late complications included granulation tissue formation, tracheocutaneous fistula, tracheomalacia, recurrent tube obstruction or displacement, recurrent respiratory tract infections, laryngotracheal stenosis, and swallowing difficulties.

Decannulation was defined as the permanent removal of the tracheostomy tube and was considered the ultimate goal of airway management for many patients. Decisions regarding decannulation were made by multidisciplinary consensus, based on resolution of the primary indication, demonstration of adequate airway patency on endoscopic evaluation, stable spontaneous respiration without RS, and normal swallowing function. All decannulations were performed during hospitalization with continuous monitoring for a minimum of 48 h.

Patients were followed longitudinally after tracheotomy until the last available clinical contact recorded in the hospital medical records. The follow-up period for all patients included in the study extended through 31 December 2024. Data were collected from inpatient records, outpatient clinic visits, and available hospital documentation. The duration of follow-up was calculated from the date of tracheotomy to the last documented follow-up visit, decannulation, death, or last available clinical record, whichever occurred first.

For patients who underwent decannulation, the time to decannulation was recorded. Mortality was recorded as all-cause mortality and included any death occurring during the follow-up period, regardless of underlying cause. Deaths were recorded as a binary outcome (alive vs. deceased at last follow-up). Patients were censored at the time of the last available clinical follow-up.

### 2.6. Statistical Analysis

Statistical analysis was performed using IBM SPSS Statistics, version 25.0 (IBM Corp., Armonk, NY, USA). Descriptive statistics were used to summarize patient characteristics, procedural variables, and clinical outcomes. Continuous variables were expressed as medians with interquartile ranges (IQRs), and categorical variables as counts and percentages. Comparative analyses between the AO and RS groups were conducted using the Mann–Whitney U test for continuous variables because of non-normal data distribution, and the Chi-square or Fisher’s exact test, as appropriate, for categorical variables. To identify independent predictors of decannulation and mortality after pediatric tracheotomy, multivariable logistic regression models were constructed. Variables included in the models were selected based on clinical relevance and univariable analysis results. The following covariates were entered into the models: age and indication for tracheotomy, urgency of the procedure, sex, and presence of comorbidities. Adjusted odds ratios (ORs) with corresponding 95% confidence intervals (CIs) were calculated. A *p*-value < 0.05 was considered statistically significant.

## 3. Results

### 3.1. Temporal Trends

**Figure 1:**
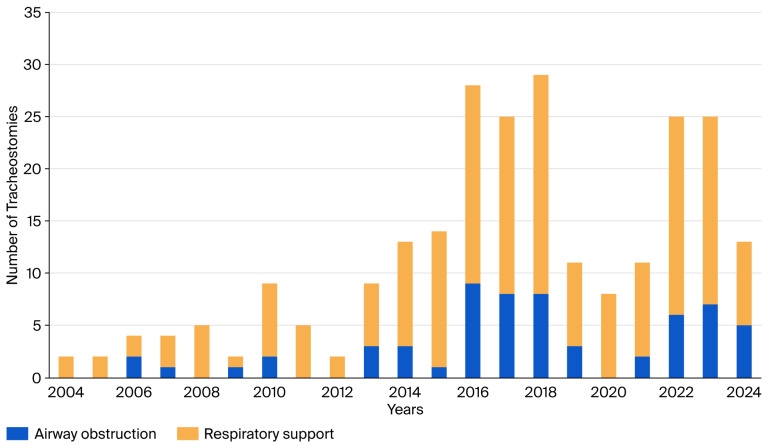
Annual number of pediatric tracheotomies between 2004 and 2024 stratified by indication group.

A total of 246 pediatric tracheotomies were performed over the 21-year study period. The annual number of procedures varied, with relatively low and stable counts during the first decade (2004–2013), averaging approximately 4.4 tracheotomies per year. A marked increase in the number of tracheotomies was observed in the second decade (2014–2024), with a peak of 29 procedures in 2018. Specifically, 202 tracheotomies were performed during 2014–2024 compared to 44 during 2004–2013, reflecting a substantial rise in procedural volume over time (Figure 1).

When stratified by indication, RS-related tracheotomies represented the predominant indication throughout the study period and were the main driver of the observed increase in total case volume. In contrast, AO-related tracheotomies remained relatively stable over time, with smaller fluctuations and no clear upward trend. In later years, particularly after 2015, RS cases accounted for the majority of procedures, reflecting a shift in clinical indication patterns over time.

### 3.2. Patient Characteristics

The study population comprised 137 males (55.7%) and 109 females (44.3%), yielding a male-to-female ratio of 1.26:1. The mean age at the time of tracheotomy was 46 months (3.8 years), ranging from 1 day to 17 years and 10 months. More than half of the patients (54.1%) underwent tracheotomy before the age of 1 year, including 121 infants (29 days to 1 year old; 49.2%) and 12 neonates (0–28 days old; 4.9%). Regarding the nature of the procedure, 225 tracheotomies (91.5%) were elective, while 21 (8.5%) were performed as emergencies (Table 1).

From all included patients, 61 (24.8%) were classified in the airway obstruction (AO) group and 185 (75.2%) in the respiratory support (RS) group. Patients in the RS group were significantly older compared with those in the AO group (*p* < 0.001). Infants (29 days–1 year) represented the largest proportion of the total cohort (49.2%), followed by children aged 1–3 years (17.5%), children aged 4–12 years (17.1%), adolescents (11.4%), and neonates (4.9%). No statistically significant difference was found in gender distribution between the groups. A significantly higher proportion of emergency tracheotomies was performed in the AO group than in the RS group (*p* < 0.001). Elective tracheotomy and intraoperative intubation were significantly more frequent in the RS group (both *p* < 0.001).

### 3.3. Distribution of Primary Indications

Regarding primary indications, respiratory support was the most common reason for tracheotomy (*n* = 185; 75.2%), while airway obstruction accounted for a smaller proportion of cases (*n* = 61; 24.8%). Among children undergoing tracheotomy for respiratory support, neurological disorders represented the predominant indication, affecting 138 patients (56.1% of the total sample) (Table 2). Within this subgroup, encephalopathies were the leading category, observed in 86 cases. Cardiopulmonary diseases were the second most frequent indication, comprising 30 patients (16.2%). The main etiology within this category was complex congenital heart defects, followed by chronic lung disease.

Among children with airway obstruction, laryngotracheal stenosis was the leading cause, identified in 51 patients (83.6%). The majority of these cases were acquired stenoses, while congenital stenosis accounted for 11 patients. Craniofacial anomalies were responsible for obstruction in 10 patients (16.4%), with Pierre Robin sequence being the most frequent anomaly in this group. Other less common causes included head and neck tumors, vocal cord paralysis, and laryngotracheomalacia.

These findings highlight the predominance of chronic and complex conditions in contemporary pediatric tracheotomy practice, reflecting advances in intensive care and improved long-term survival among children with severe comorbidities.

### 3.4. Comparative Analysis of Pediatric Tracheotomy Outcomes by Indication Group

Complication rates did not differ significantly between the two groups (AO: 16.4% vs. RS: 21.1%; *p* = 0.295) (Table 3). Overall, decannulation was achieved in 20 patients (8.1%) of the entire cohort, and decannulation success was significantly higher in the AO group (16.4%) than in the RS group (5.4%; *p* = 0.012). Although not statistically significant, mortality was higher in the RS group (AO: 14.8% vs. RS: 23.8%; *p* = 0.154).

Among patients with AO and those requiring RS, there were no statistically significant differences in follow-up duration, time to decannulation, or time to death. Median follow-up was 34.10 months (IQR 8.77–92.67) in the AO group and 55.10 months (IQR 17.34–101.70) in the RS group (*p* = 0.241). Median time from tracheotomy to decannulation was 42.51 months (IQR 23.24–76.72) versus 41.55 months (IQR 13.11–52.61), respectively (*p* = 0.684). Median time to death was 15.90 months (IQR 3.40–56.81) in the airway obstruction group and 30.72 months (IQR 6.13–50.43) in the respiratory support group (*p* = 0.265) (Table 4).

Multivariable logistic regression analysis showed that airway obstruction as the indication for tracheotomy was associated with higher odds of decannulation compared with respiratory support (OR 0.28, 95% CI 0.10–0.76, *p* = 0.013). Female sex was associated with lower odds of death (OR 0.53, 95% CI 0.28–0.99, *p* = 0.048). Age, emergency procedure status, and comorbidity burden were not significantly associated with either decannulation or mortality (Table 5).

## 4. Discussion

This 21-year study provides insights into shifts in the epidemiology and clinical indications for pediatric tracheotomy. Although smaller than registry-based studies, our cohort provides detailed longitudinal clinical data from a tertiary referral center in Serbia. While single-institution studies may reflect local practice patterns, they offer detailed clinical characterization that complements large national databases, which are better suited to evaluating population-level trends in incidence, indications, and outcomes [14,15,16,17,18,19,20,21,22,23,24].

Our study showed a marked increase in tracheotomies over time, with most procedures performed in the second decade. This trend likely reflects increased institutional procedural volume rather than a true population-level epidemiological change. It may also be influenced by evolving referral patterns, expanded pediatric intensive care capacity, improved survival of medically complex children, and better documentation practices. The predominance of tracheotomy for respiratory support in our cohort is consistent with contemporary trends reported in the literature and reflects the growing need for long-term ventilatory support among critically ill children and those with neurological and chronic cardiopulmonary conditions [13,15,21,22,25,26]. 

Technological advances in surgical, anesthetic, and postoperative care have improved procedural safety, while evolving disease patterns have influenced tracheotomy indications and outcomes.

Nearly half of the patients in our cohort were infants, with smaller proportions of newborns, children, and adolescents, and the mean age at tracheotomy was 46 months. Consistent with global data, a bimodal age distribution was observed, with the highest incidence in infants (0–1 year) and a secondary, smaller peak in older children (14–18 years), reflecting the broad age spectrum in which tracheotomy is required [2,25,26,27]. There was a slight male predominance, with no significant difference between the AO and RS groups, aligning with international reports. 

More than 90% of procedures were performed electively under intraoperative intubation, with RS as the leading indication. Overall procedural characteristics differed markedly between the AO and RS groups. In emergency cases, tracheotomy was performed as the first and only means of securing the airway, whereas elective procedures were performed under controlled conditions. Consistent with the acute nature of AO, most tracheotomies in this group were performed emergently, while RS cases were predominantly elective [24,25]. Children undergoing tracheotomy for AO were significantly younger and more likely to require urgent intervention compared with those undergoing tracheotomy for RS.

Complication rates were comparable between AO and RS groups, while decannulation outcomes differed significantly in favor of AO. These results may suggest that although AO often necessitates earlier intervention, favorable long-term airway outcomes remain achievable. In contrast, children requiring tracheotomy for RS appear to face greater challenges with eventual decannulation.

Decannulation success in this cohort was low compared with published literature, but significantly higher in the AO group, consistent with the influence of underlying etiology on long-term airway recovery [25,27]. These findings reinforce that patient selection and underlying etiology are the most important determinants of decannulation success, highlighting the need for individualized management strategies.

These patients require highly specialized multidisciplinary management, including home ventilation services, structured caregiver education, regular airway surveillance, and coordinated outpatient follow-up. Such long-term dependence also places a considerable psychological, organizational, and financial burden on families and healthcare systems, particularly in resource-limited settings.

Overall complication rates were consistent with previously reported literature, with no significant difference between indication groups [4,5,23]. This finding may reflect institutional factors, including patient selection, surgical expertise, and perioperative care standards. Early complications occurred in 7 patients (3%), consisting of three cases of cannula size–related ventilation difficulties and four accidental decannulations within the first postoperative week, one of which resulted in subcutaneous emphysema during recannulation attempts. This rate compares favorably with published reports, where early complication rates of up to 11% have been described [14,26], and may reflect the effectiveness of immediate postoperative monitoring and management protocols. Late complications were observed in 42 patients (17%), with stomal infections and stomal stenosis being the most frequent. The observed late complication rate likewise falls below the upper range reported in the literature (up to 63%) [14,27]. The relatively low observed complication rate should be interpreted cautiously due to the retrospective design of the study and the inherent possibility of underreporting, particularly for minor complications during the earlier years of the study period. Furthermore, changes in documentation practices over the 21-year study period may have influenced complication capture and reporting. 

Post-tracheotomy mortality did not differ significantly between the AO and RS groups. This finding is consistent with international reports, which indicate low tracheotomy-associated mortality (0–5.9%) but higher overall mortality in patients with complex medical conditions [4,14,15,16,21,23]. No deaths were directly attributed to tracheotomy, including AO-related complications or accidental decannulation. Observed mortality was most likely related to the underlying disease burden, and the low procedure-related mortality may suggest effective perioperative management despite the high-risk population.

Optimal management of children undergoing tracheotomy requires multidisciplinary tracheotomy care programs that include structured caregiver education, coordinated outpatient follow-up, and reliable access to pediatric home ventilation services. Such comprehensive care pathways are essential to reduce preventable complications and to support long-term survival and quality of life in this complex patient population [28,29,30]. 

This study benefits from a long-term 21-year observation period, providing valuable insights into trends in pediatric tracheotomy indications, procedural characteristics, and outcomes. Detailed clinical data, including complications, decannulation success, mortality, follow-up duration, and time to decannulation, allowed a comprehensive evaluation, while subgroup comparisons between AO and RS patients highlighted meaningful differences in age, urgency, and long-term outcomes. Conducting the study at a single tertiary institution ensured consistency in surgical technique, anesthetic management, and postoperative care. 

Despite these strengths, the findings should be interpreted with caution. The single-center design and relatively modest sample size may limit generalizability, while the retrospective nature of the study restricts control over potential confounding factors, including the severity of underlying comorbidities. Further multicenter studies with more granular perioperative data are needed to better identify independent predictors of outcome.

A limitation of this study is the lack of standardized severity grading of complications (e.g., Clavien–Dindo classification), as retrospective documentation over the long study period did not allow consistent application of such systems, highlighting the need for future prospective studies to incorporate standardized complication grading and improve comparability across reports.

Although surgical tracheotomy was performed using a standardized open technique throughout the study period, minor variations in tracheal incision, including selective use of a Björk flap, occurred depending on surgeon preference and intraoperative conditions. These variations may have influenced local outcomes but could not be systematically evaluated in this retrospective analysis.

The study is further limited by the absence of a standardized protocol for decannulation decisions, and variability in clinical practice may have influenced both timing and likelihood of decannulation. In addition, reasons for non-decannulation and data on unsuccessful decannulation attempts were incompletely documented in a subset of patients. Follow-up duration also varied across the 21-year study period, which may have affected observed decannulation rates, particularly among patients treated in later years with shorter observation times. At the last follow-up, many patients remained tracheotomy-dependent, while others died before decannulation or continued to require long-term tracheotomy care.

An important limitation in interpreting our findings is the inherent clinical heterogeneity between the two groups, which differ substantially in age distribution, urgency of intervention, intraoperative airway status, and underlying disease severity, all of which may influence complications, decannulation, and mortality. Therefore, observed differences between groups should be interpreted as unadjusted associations reflecting real-world clinical variation rather than evidence of independent causal effects. Although multivariable regression analyses were performed for selected outcomes, residual confounding cannot be excluded due to the strong interdependence between variables. In particular, intraoperative intubation status is closely linked to the underlying indication and clinical presentation.

Furthermore, as a tertiary referral center for medically complex children, our institution likely manages a disproportionate number of patients with severe neurological and cardiopulmonary comorbidities requiring long-term respiratory support. This referral pattern may have resulted in selection bias and limited the representativeness of the cohort relative to the broader pediatric tracheotomy population.

Due to the retrospective nature of the study and the long observation period, cause-specific mortality (e.g., procedure-related vs disease-related) could not be consistently and reliably determined and was therefore not analyzed separately. In addition, limited etiological and genetic data prevented more detailed subgroup analyses, including exploration of factors potentially contributing to the observed male predominance.

Finally, although the impact of pediatric tracheotomy on patients and families is well recognized, this study did not include a formal assessment of psychosocial outcomes, caregiver burden, or quality of life using validated instruments. Future prospective studies should address these important patient- and family-centered outcomes.

## 5. Conclusions

Pediatric tracheotomy has evolved over the past two decades, with a marked increase in procedures primarily driven by the need for long-term respiratory support. RS remains the leading indication, while AO is more often associated with younger age and urgent intervention. Procedural safety is high, with low overall complication rates, and decannulation success is higher in children with AO compared to those requiring prolonged ventilatory support. Mortality is largely determined by the severity of underlying conditions rather than the procedure itself. These findings underscore the importance of individualized management and multidisciplinary care in optimizing outcomes for pediatric tracheotomy patients. Based on our results, several practice-oriented improvements can be recommended. The development and implementation of standardized decannulation protocols is essential to reduce variability in clinical decision-making and to ensure consistent, criteria-based assessment of readiness for decannulation. Optimal management requires multidisciplinary tracheotomy care programs that include structured caregiver education (covering suctioning techniques, emergency airway management, and ventilator use), coordinated outpatient follow-up, and reliable access to pediatric home ventilation services.

## Figures and Tables

**Table 1:** Demographic and perioperative characteristics of pediatric patients undergoing tracheotomy, stratified by indication group.

Variable	Airway Obstruction *N* (%)	Respiratory Support *n* (%)	Total *n* (%)	*p* Value
Age				<0.001
Newborn (0–28 days old)	11 (91.7)	1 (8.3)	12 (4.9%)	
Infant (29 days–1 year old)	31 (25.6)	90 (74.4)	121 (49.2%)	
Child (1–3 years)	10 (23.3)	33 (76.6)	43 (17.5%)	
Child (4–12 years old)	4 (9.5)	38 (90.5)	42 (17.1%)	
Adolescent (13–18 years old)	5 (17.9)	23 (82.1)	28 (11.4%)	
Gender				0.151
Male	30 (21.9)	107 (78.1)	137 (55.7%)	
Female	31 (28.4)	78 (71.6)	109 (44.3%)	
Procedure urgency				<0.001
Emergency	18 (85.7)	3 (14.3)	21 (8.5%)	
Elective	43 (19.1)	182 (80.9)	225 (91.5%)	
Intraoperative ventilation				<0.001
Intubated	45 (19.8)	182 (80.2)	227 (92.3%)	
Non-intubated	16 (84.2)	3 (15.8)	19 (7.7%)	

**Table 2:** Indications for tracheotomy.

Airway Obstruction	*N* = 61
Laryngotracheal obstruction	51 (83.6)
Congenital laryngotracheal stenosis	11 (18.0)
Acquired laryngotracheal stenosis	17 (27.9)
Head/neck tumor	13 (21.3)
Vocal cord paralysis	7 (11.5)
Laryngotracheomalacia	3 (4.9)
Craniofacial anomalies	10 (16.4)
Pierre Robin syndrome	8 (13.1)
Treacher Collins syndrome	1 (1.6)
Goldenhar syndrome	1 (1.6)
**Respiratory Support**	***N* = 185**
Cardio-pulmonary diseases	30 (16.2)
Congenital heart disease	23 (12.4)
Lung disease	2 (1.1)
Bronchopulmonary dysplasia	5 (2.7)
Neurological diseases	138 (74.6)
Brain tumor	10 (5.4)
Encephalopathy	86 (46.5)
Neuromuscular disease	39 (21.1)
Trauma	3 (1.6)
Genetic conditions and birth defects	11 (5.9)
Larsen syndrome	1 (0.5)
Charge syndrome	4 (2.2)
Cornelia de Lange syndrome	1 (0.5)
Niemann-Pick disease	1 (0.5)
Gaucher disease	1 (0.5)
Multiple malformation syndrome	1 (0.5)
Omphalocele	1 (0.5)
Total colonic aganglionosis	1 (0.5)
Respiratory support due to other reasons	6 (3.2)
Acute respiratory infection	3 (1.6)
Chronic respiratory failure	1 (0.5)
Kidney tumor	1 (0.5)
Childhood leukaemia	1 (0.5)

Percentages are calculated within each indication group (airway obstruction *N* = 61; respiratory support *N* = 185).

**Table 3:** Outcomes following tracheotomy by indication.

Variable	Airway Obstruction *n* (%)	Respiratory Support *n* (%)	*p*-Value
Complications			0.295
Yes	10 (16.4)	39 (21.1)	
No	51 (83.6)	146 (78.9)	
Successfully decannulated			0.012
Yes	10 (16.4)	10 (5.4)	
No	51 (83.6)	175 (94.6)	
Death			0.154
Yes	9 (14.8)	44 (23.8)	
No	52 (85.2)	141 (76.2)	

**Table 4:** Follow-up and time-to-event outcomes after pediatric tracheotomy.

	Airway Obstruction 61 (24.8%)	Respiratory Support 185 (75.2%)	*p* Value
Follow–up months, median (IQR)	34.10 (8.77–92.67)	55.10 (17.34–101.70)	0.241
Time to decannulation *, months, median (IQR)	42.51 (23.24–76.72)	41.55 (13.11–52.61)	0.684
Time to death **, median (IQR)	15.90 (3.40–56.81)	30.72 (6.13–50.43)	0.265

* among decannulated patients, ** among deceased patients.

**Table 5:** Multivariable logistic regression analysis of factors associated with decannulation and mortality after pediatric tracheotomy.

	Decannulation		Death	
	OR (95% CI)	*p* Value	OR (95% CI)	*p* Value
Age at tracheotomy	0.96 (0.87–1.07)	0.460	0.96 (0.91–1.03)	0.244
Female sex	0.51 (0.19–1.33)	0.170	0.53 (0.28–0.99)	0.048
Emergency vs. elective	0.49 (0.09–2.53)	0.394	1.17 (0.32–4.26)	0.822
AO vs. RS	0.28 (0.10–0.76)	0.013	2.13 (0.87–5.23)	0.098
Comorbidity	1.17 (0.24–5.57)	0.846	1.76 (0.70–4.43)	0.229

OR: odds ratio; CI: confidence interval.

## Data Availability

The data presented in this study are available on reasonable request from the corresponding author. The data are not publicly available due to ethical and privacy restrictions involving pediatric patients.

## Abbreviations

The following abbreviations are used in this manuscript:

AO	Airway Obstruction
RS	Respiratory Support
BPD	Bronchopulmonary Dysplasia
ARI	Acute Respiratory Infection
CHR	Chronic Respiratory Failure
